# Iron-Sulfur Cluster Repair Contributes to Yersinia pseudotuberculosis Survival within Deep Tissues

**DOI:** 10.1128/IAI.00533-19

**Published:** 2019-09-19

**Authors:** Kimberly M. Davis, Joanna Krupp, Stacie Clark, Ralph R. Isberg

**Affiliations:** a Department of Molecular Biology and Microbiology, Tufts University School of Medicine, Boston, Massachusetts, USA; b Graduate Program in Molecular Microbiology, Tufts University School of Medicine, Boston, Massachusetts, USA; University of California—San Diego School of Medicine

**Keywords:** *Yersinia pseudotuberculosis*, iron-sulfur cluster repair, nitric oxide

## Abstract

To successfully colonize host tissues, bacteria must respond to and detoxify many different host-derived antimicrobial compounds, such as nitric oxide (NO). NO has direct antimicrobial activity through attack on iron-sulfur (Fe-S) cluster-containing proteins. NO detoxification plays an important role in promoting bacterial survival, but it remains unclear if repair of Fe-S clusters is also important for bacterial survival within host tissues. Here we show that the Fe-S cluster repair protein YtfE contributes to the survival of Yersinia pseudotuberculosis within the spleen following nitrosative stress. Y. pseudotuberculosis forms clustered centers of replicating bacteria within deep tissues, where peripheral bacteria express the NO-detoxifying gene *hmp. ytfE* expression also occurred specifically within peripheral cells at the edges of microcolonies. In the absence of *ytfE*, the area of microcolonies was significantly smaller than that of the wild type (WT), consistent with *ytfE* contributing to the survival of peripheral cells. The loss of *ytfE* did not alter the ability of cells to detoxify NO, which occurred within peripheral cells in both WT and Δ*ytfE* microcolonies. In the absence of NO-detoxifying activity by *hmp*, NO diffused across Δ*ytfE* microcolonies, and there was a significant decrease in the area of microcolonies lacking *ytfE*, indicating that *ytfE* also contributes to bacterial survival in the absence of NO detoxification. These results indicate a role for Fe-S cluster repair in the survival of Y. pseudotuberculosis within the spleen and suggest that extracellular bacteria may rely on this pathway for survival within host tissues.

## Abstract (precis)

To successfully colonize host tissues, bacteria must respond to and detoxify many different host-derived antimicrobial compounds, such as nitric oxide (NO). NO has direct antimicrobial activity through attack on iron-sulfur (Fe-S) cluster-containing proteins. NO detoxification plays an important role in promoting bacterial survival, but it remains unclear if repair of Fe-S clusters is also important for bacterial survival within host tissues.

## INTRODUCTION

Nitric oxide (NO) is a diffusible gas that has a wide range of physiological functions within mammals (1, 2). The effects of NO are tissue concentration dependent, as it promotes vasodilation, cell proliferation, and cell differentiation at low concentrations (3, 4), while high concentrations drive apoptosis and defense against bacteria, fungi, and parasites (5–8). NO is produced by three different nitric oxide synthase (NOS) isoforms within mammalian tissues: neuronal NOS (nNOS), endothelial NOS (eNOS), and inducible NOS (iNOS). nNOS and eNOS are expressed at low levels by endothelial and neuronal cells, respectively (2). In contrast, iNOS is expressed by a wide range of cell types, specifically in response to NF-κB-dependent sensing, and is responsible for the high levels of NO produced during infection (1, 9).

NO has direct antimicrobial activity and can also react with reactive oxygen species (ROS) to produce additional toxic compounds, such as peroxynitrite. NO may be bacteriostatic, while peroxynitrite is known to have direct bactericidal activity (10). NO antibacterial activity occurs through nitrosylation of iron-sulfur (Fe-S) cluster-containing proteins, which play critical roles in cellular respiration, DNA synthesis, and gene regulation. NO also targets heme groups, reactive thiols, and tyrosyl radicals and can cause DNA damage (1, 10). One of the global regulators in Escherichia coli that has been shown to be inactivated by NO is NsrR (11), which regulates the response to nitrosative stress and is also associated with the oxidative stress response (12–14). Nitrosylation of the NsrR-associated Fe-S cluster relieves the repression of at least 60 genes in E. coli (15–18). Included in this regulon are the *hmp* gene, which encodes a flavohemoglobin that detoxifies NO (19, 20), and YtfE (also known as repair of iron centers [RIC] in E. coli), which functions to repair Fe-S clusters following NO damage (13, 17). Repair of Fe-S cluster proteins by YtfE can eliminate the need for new Fe-S cluster biogenesis when Fe availability is limited (13, 21, 22). A variety of studies argue that YtfE contributes to bacterial survival within host cells, but it remains unclear if YtfE contributes to the survival of extracellular pathogens replicating within host tissues and if YtfE contributes to survival following exposure to NO (23–25).

Yersinia pseudotuberculosis is an oral pathogen that is typically contained within intestinal tissues and gut-associated lymphoid tissues but has the capacity to spread systemically in susceptible individuals (26–29). Following bloodstream access, Y. pseudotuberculosis colonizes deep tissue sites where individual bacteria replicate to form clonal microcolonies (30–32). Neutrophils, monocytes, and macrophages are recruited to sites of bacterial replication, which are kept at bay by *Yersinia*, primarily via substrates of the bacterial type III secretion system (33–35). Recruited monocytes and macrophages produce NO, which diffuses across a layer of neutrophils and is inactivated at the periphery of the microcolony by bacteria expressing Hmp, preventing diffusion of NO into the interior of the microcolony (32). The consequences of the selective attack of NO on peripheral bacteria are unclear, although it is likely that nitrosative stress may slow their growth. Additional members of the nitrosative stress response, such as YtfE, may also be required in peripheral cells to ensure their survival.

Bacteria responding to reactive nitrogen species (RNS) appear to recover and remain viable, consistent with members of the NsrR regulon cooperating to repair NO-mediated damage in peripheral bacteria within microcolonies. Two of the members of the E. coli NsrR regulon, *hmp* and *ytfE*, are upregulated in the bubonic plague model of Yersinia pestis infection and during Peyer’s patch colonization by Y. pseudotuberculosis (36, 37). The similar expression patterns of *hmp* and *ytfE* may suggest that both genes are also members of the NsrR regulon in *Yersinia*, and both could contribute to microbial fitness during microcolony growth. Additionally, very few studies have explored the role of Fe-S cluster repair during infection with extracellular bacteria. Here we show that *ytfE* contributes to the survival of extracellular bacteria, specifically through upregulation of *ytfE* within peripheral cells of Y. pseudotuberculosis microcolonies.

## RESULTS

### Y. pseudotuberculosis *ytfE* expression is regulated by NsrR and occurs within peripheral cells during growth in the spleen.

**FIG 1:**
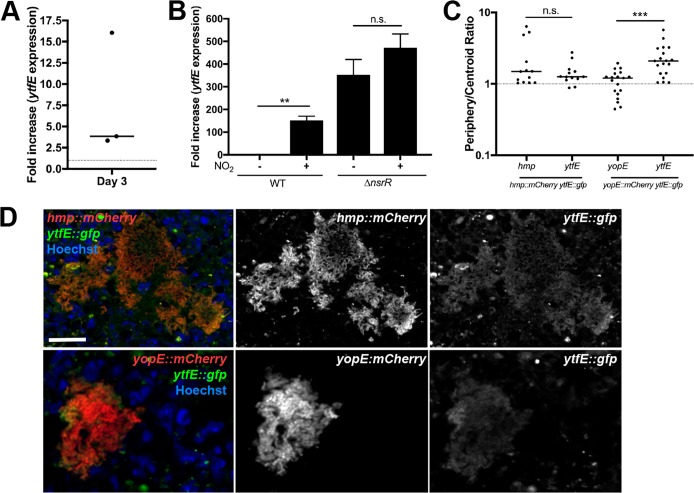
*ytfE* expression is regulated by NsrR and occurs within peripheral cells. (A) C57BL/6 mice were inoculated intravenously (i.v.) with 10^3^ WT Y. pseudotuberculosis bacteria, and spleens were harvested at day 3 postinoculation (p.i.). Bacterial transcripts were isolated from splenic tissue, transcript levels were quantified by qRT-PCR relative to 16S, and the fold increase in *ytfE* transcript levels is shown relative to the inoculum. Each dot represents an individual mouse. (B) Nitrogen stress was induced (+) in cultures of WT and Δ*nsrR* strains and compared to that in untreated cultures (−). The *ytfE* transcript levels are expressed relative to 16S, and the fold increase is relative to the average level in untreated WT cultures. Each column shows data for 5 biological replicates (means and standard errors of the means [SEM]). (C) C57BL/6 mice were inoculated i.v. with the WT *hmp*::*mCherry ytfE*::*gfp* strain or the WT *yopE*::*mCherry ytfE*::*gfp* strain, and spleens were harvested at day 3 p.i. for fluorescence microscopy. The fluorescence signal intensity was determined within the same peripheral and centroid cells for *hmp* and *ytfE* reporters or *yopE* and *ytfE* reporters and divided to generate the periphery/centroid ratio (4 mice/group). Dots indicate individual microcolonies. (D) Representative images of *hmp*::*mCherry* and *ytfE*::*gfp* reporters (top) or *yopE*::*mCherry* and *ytfE*::*gfp* reporters (bottom). Merge and single-channel images are shown. Bar, 20 μm. Statistical analysis was performed by a Wilcoxon matched-pairs test (**, *P* < 0.01; ***, *P* < 0.001; n.s., not significant).

The *ytfE* gene is known to be a member of the NsrR regulon in a number of bacterial species (13, 14, 38), so *ytfE* is expected to be repressed by NsrR in Y. pseudotuberculosis. We also expected that *ytfE* is transcribed during Y. pseudotuberculosis growth within the mouse spleen, as the NsrR-regulated *hmp* gene is expressed in this tissue (23–25, 32, 36). To determine if Y. pseudotuberculosis *ytfE* is expressed during splenic growth, C57BL/6 mice were intravenously (i.v.) challenged with bacteria, and bacterial RNA was isolated at day 3 postinoculation (p.i.). Based on reverse transcription-quantitative PCR (qRT-PCR) analysis, *ytfE* transcript levels increased within the mouse spleen relative to the inoculum culture grown in the absence of an NO-generating system, with marked mouse-to-mouse variation (Fig. 1A) (32). To determine if *ytfE* expression is NsrR dependent, we compared *ytfE* transcription levels in wild-type (WT) and Δ*nsrR* strains in the presence and absence of nitrogen stress imparted by acidified nitrite (NO_2_). The transcription of *ytfE* increased by over 100-fold in the WT strain with the addition of nitrogen stress (Fig. 1B). The transcription level of *ytfE* was high in the Δ*nsrR* strain in either the presence or absence of nitrogen stress, indicating that NsrR negatively regulates *ytfE* transcription. A slight increase in *ytfE* expression in the Δ*nsrR* strain with nitrogen stress suggests that additional pathways may regulate *ytfE* expression.

Hmp, another member of the NsrR regulon, is specifically expressed on the periphery of Y. pseudotuberculosis microcolonies during splenic growth (32). At the transcriptional level, this is associated with considerable variation in expression levels between individual mice. Variation is likely due to the presence of different-size microcolonies within different organs, as distinct peripheral expression of *hmp* is visualized in large microcolonies, and smaller microcolonies are more homogenous. The variable *ytfE* expression levels in the mouse and NsrR dependence are reminiscent of *hmp* expression, indicating that there could be a link between spatial expression and intermouse variation. To determine if *ytfE* and *hmp* have similar expression patterns during growth in the spleen, mice were intravenously inoculated with a WT Y. pseudotuberculosis strain containing *hmp*::*mCherry* (chromosomal integration of *mCherry* downstream of *hmp*) and *ytfE*::*gfp* (chromosomal integration of *gfp* downstream of *ytfE*). Microcolonies were visualized within the spleen using fluorescence microscopy, and *ytfE* and *hmp* reporter signals were quantified within the same cells at the center and periphery of the microcolonies (see Materials and Methods). The *hmp* reporter signal increased within individual cells at the periphery relative to cells at the centroid of microcolonies, which generated a ratio value greater than 1, consistent with *hmp* peripheral expression (Fig. 1C). The *ytfE* signal was dim relative to that of *hmp* but also increased at the periphery of microcolonies relative to the centroid. The periphery/centroid signal intensity ratio value for *ytfE* was similar to that for *hmp*, indicating that *ytfE* was expressed at the periphery of microcolonies (Fig. 1C). To confirm that the dim *ytfE* signal was not due to mCherry fluorescence detected in the *gfp* channel, experiments were also performed in a WT strain containing *yopE*::*mCherry* and *ytfE*::*gfp. yopE* and *ytfE* exhibited distinct reporter patterns, indicating that the detected *gfp* signal was due to *ytfE* reporter expression (Fig. 1C and D). There was significant overlap in the *hmp* and *ytfE* signals within these images, and based on NsrR-dependent regulation of both genes, it is likely that *hmp* and *ytfE* were expressed in the same cells (Fig. 1D).

### *ytfE* contributes to the virulence of Y. pseudotuberculosis in the spleen.

**FIG 2:**
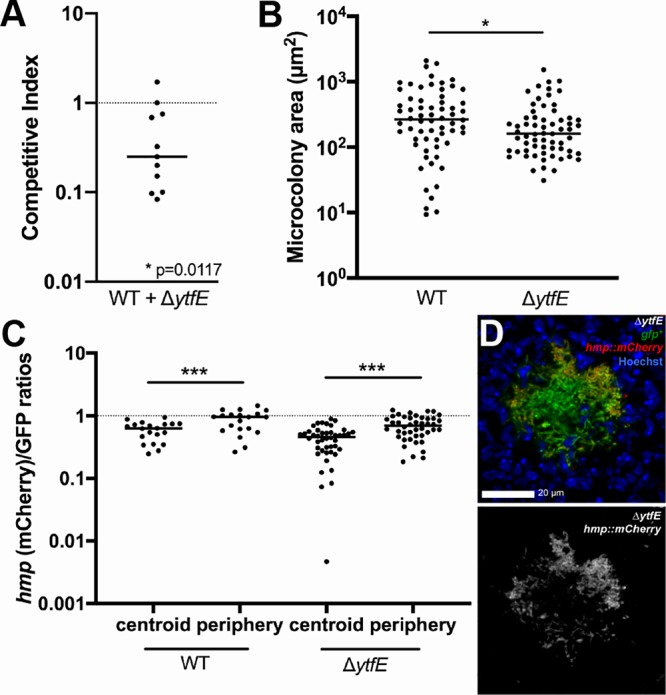
*ytfE* contributes to growth in the spleen. (A) Coinfection with the WT and Δ*ytfE* strains. C57BL/6 mice were inoculated i.v. with an equal mixture of mCherry^+^ (*yopE*::*mCherry*) WT and GFP^+^ Δ*ytfE* strains, and spleens were harvested at day 3 p.i. The competitive index is the ratio of Δ*ytfE*/WT CFU in the spleen at day 3 divided by ratio of Δ*ytfE*/WT CFU in the inoculum. Dots indicate individual mice. The dotted line indicates equal fitness, with replicates completed on two separate days. (B) Quantification of WT and Δ*ytfE* microcolony areas (square micrometers) from the coinfection in panel A (see Materials and Methods), in 11 mice. (C) C57BL/6 mice were inoculated i.v. with WT GFP^+^ *hmp*::*mCherry* or Δ*ytfE* GFP^+^ *hmp*::*mCherry* strains, and spleens were harvested at day 3 p.i. for fluorescence microscopy. Reporter signals were quantified within peripheral and centroid cells, and the *hmp* reporter signal was divided by the GFP signal. (D) Representative images of a Δ*ytfE* GFP^+^ *hmp*::*mCherry* microcolony, stained with Hoechst stain to detect host nuclei. Merge and *hmp* single-channel images are shown. Statistical analyses were performed by a Wilcoxon signed-rank test, compared to a value of 1 (A); a Mann-Whitney test (B); or a Wilcoxon matched-pairs test (C) (*, *P* < 0.05; ***, *P* < 0.001).

YtfE repairs Fe-S clusters damaged by NO, and there appears to be sufficient NO at the periphery of splenic microcolonies to allow synthesis of this protein and to promote repair within this subpopulation of bacteria. To determine if the loss of *ytfE* alters the overall fitness of Y. pseudotuberculosis during growth within the spleen, we constructed a Y. pseudotuberculosis strain that lacks the *ytfE* gene (Δ*ytfE*) and harbors a constitutive *gfp*-expressing plasmid, to visualize growth within the spleen. To compare differences in relative fitness, mice were infected intravenously with equal amounts of WT mCherry-positive (mCherry^+^) and Δ*ytfE* green fluorescent protein-positive (GFP^+^) strains, and spleens were harvested at day 3 p.i., a late stage of infection in this model, to determine the competitive index (CI) by CFU and quantify microcolony areas within the same animals. At day 3 p.i., the median competitive index was significantly less than 1, indicating a lowered fitness of the Δ*ytfE* strain relative to the WT strain (*P* = 0.0115) (Fig. 2A). The areas of individual microcolonies within these coinfected tissues were also visualized and quantified by fluorescence microscopy in 11 mice. The Δ*ytfE* microcolonies were significantly smaller than WT microcolonies within the same organs, indicating that *ytfE* contributes to the survival of Y. pseudotuberculosis in the spleen, presumably due to lowered fitness of the bacterial population located at the periphery of the microcolonies (Fig. 2B).

Since YtfE could directly repair the Fe-S cluster of NsrR, the absence of *ytfE* could alter the expression of the NsrR regulon, by resulting in heightened expression within peripheral cells. To determine if microcolonies from the Y. pseudotuberculosis Δ*ytfE* strain have sustained expression of the NsrR regulon relative to the WT strain, we infected mice intravenously with WT GFP^+^ *hmp*::*mCherry*- or Δ*ytfE* GFP^+^ *hmp*::*mCherry*-integrated *hmp* reporter strains, and spleens were harvested at day 3 p.i. to visualize reporter expression by fluorescence microscopy. Similar to the data in Fig. 1D, the *hmp* reporter expression level was significantly higher at the periphery than in the centroid in the WT strain (Fig. 2C). The reporter expression pattern was very similar in Δ*ytfE* microcolonies, indicating that there was still a gradient of NO exposure in the mutant, in which Hmp activity in the peripheral population protects the central population of bacteria, and that the loss of *ytfE* did not significantly alter the expression of the NsrR regulon (Fig. 2D).

### *ytfE* contributes to bacterial survival in the absence of *hmp*.

**FIG 3:**
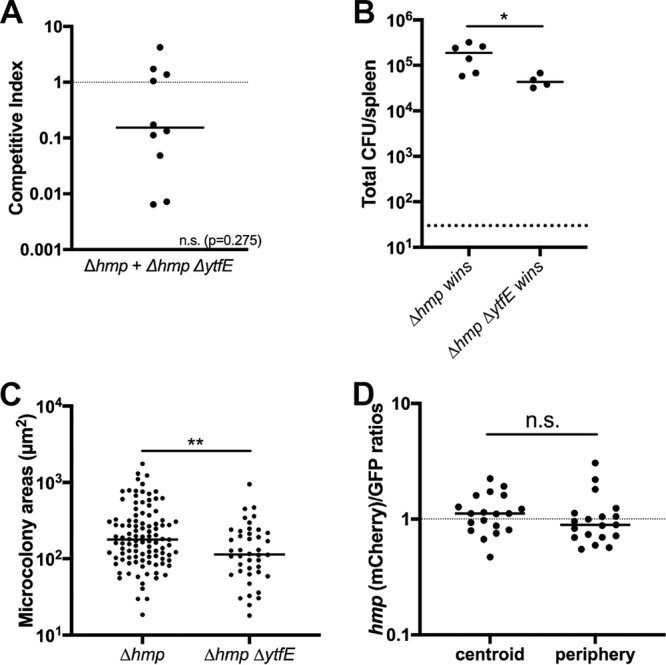
Δ*ytfE* contributes to survival in the absence of *hmp*. (A) C57BL/6 mice were inoculated i.v. with an equal mixture of *hmp* GFP^+^ and Δ*hmp* Δ*ytfE* GFP^+^ *P_hmp_*::*mCherry* strains, and spleens were harvested at day 3 p.i. The competitive index is the ratio of Δ*hmp* Δ*ytfE*/Δ*hmp* CFU in the spleen at day 3 divided by ratio of Δ*hmp* Δ*ytfE*/Δ*hmp* CFU in the inoculum. Dots indicate individual mice. The dotted line represents equal fitness. Statistical analyses were performed by a Wilcoxon signed-rank test, compared to a value of 1 (n.s., not significant). (B) Total CFU in the spleen during coinfection, when the CI was less than 1 (the Δ*hmp* strain wins) or above or equal to 1 (the Δ*hmp* Δ*ytfE* strain wins). Dots indicate individual mice. (C) Quantification of Δ*hmp* and Δ*hmp* Δ*ytfE* microcolony areas (square micrometers) from coinfection (see Materials and Methods) in 10 mice. Dots indicate individual microcolonies. (D) Reporter signals were quantified within peripheral and centroid cells in the Δ*hmp* Δ*ytfE* strain during coinfection. The *hmp* reporter signal was divided by the GFP signal. Dots indicate individual microcolonies. Statistical analyses were performed by a Mann-Whitney test (B and C) or a Wilcoxon matched-pairs test (D) (*, *P* < 0.05; **, *P* < 0.01).

YtfE contributed to the growth of Y. pseudotuberculosis microcolonies in the spleen despite expression being limited to the microcolony periphery. We were then interested in determining if YtfE-mediated repair played an important role in the context of a Δ*hmp* strain, where all bacteria in a microcolony are exposed to NO (32). To address this point, we challenged mice intravenously with Δ*hmp* GFP^+^ *P_hmp_*::*mCherry* and Δ*hmp* Δ*ytfE* GFP^+^ *P_hmp_*::*mCherry* strains, and spleens were harvested at day 3 p.i. to quantify CFU, visualize microcolony areas, and detect reporter signals by fluorescence microscopy. The Δ*hmp* Δ*ytfE* strain showed no potentiation of the defect in single-strain infections, but this defect can be observed during coinfection. The median CI value for the double mutant was below 1 but was not significantly less than 1, which suggests that the Δ*hmp* Δ*ytfE* strain may not be less fit than the Δ*hmp* strain (Fig. 3A). Interestingly, in 4 mice, the CI was at least 1, indicating that the Δ*hmp* Δ*ytfE* strain could compete efficiently with the Δ*hmp* strain in these animals. We then compared the total CFU in the organs in mice in which the Δ*hmp* strain outcompeted the Δ*hmp* Δ*ytfE* strain and in mice in which no such outcompetition took place (Fig. 3B). The total CFU were significantly lower in organs in which the Δ*hmp* strain did not outcompete the Δ*hmp* Δ*ytfE* strain, indicating that the fitness differences were suppressed in animals showing increased restriction of bacterial growth. It is also possible that the Δ*hmp* Δ*ytfE* strain had reduced levels of initial seeding within tissues. The microcolony areas were quantified within all the spleens depicted in Fig. 3A, and the areas of Δ*hmp* Δ*ytfE* microcolonies were significantly smaller than those of the Δ*hmp* strain (Fig. 3C). Δ*hmp* Δ*ytfE* microcolonies had similar *P_hmp_* reporter expression levels at the centroid and periphery, indicating that NO diffused across these centers (Fig. 3D). Together, these results confirm that *ytfE* contributes to bacterial survival in the absence of Hmp detoxifying activity.

### *ytfE* has limited effects on NO sensitivity in the absence of *hmp*.

**FIG 4:**
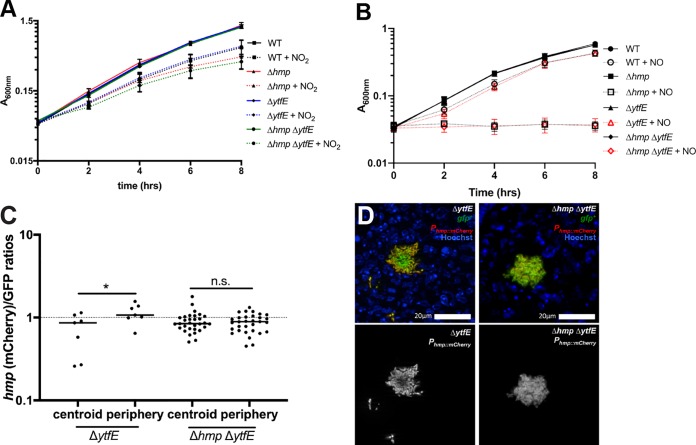
*ytfE* has limited effects on NO sensitivity in the absence of *hmp*. (A) The optical density (*A*_600_) was measured every 2 h [time (hrs)] within cultures of the indicated strains during incubation in LB at pH 5.5 in the presence (+ NO_2_) and absence of NaNO_2_. Data represent results from two independent experiments. (B) The optical density (*A*_600_) was measured every 2 h [time (hrs)] within cultures of the indicated strains during incubation in M9 minimal medium in the presence (+ NO) and absence of the NO donor DETA-NONOate. Data represent results from three independent experiments. (C) C57BL/6 mice were inoculated i.v. with the Δ*ytfE* GFP^+^ *P_hmp_*::*mCherry* or Δ*hmp ΔytfE* GFP^+^ *P_hmp_*::*mCherry* strain, and spleens were harvested at day 3 p.i. for fluorescence microscopy. Reporter signals were quantified within peripheral and centroid cells, and the *hmp* reporter signal was divided by the GFP signal. Dots represent individual microcolonies. (D) Representative images of Δ*ytfE* and Δ*hmp ΔytfE* microcolonies. Merge and *hmp* single-channel images are shown. Statistical analyses were performed by a Wilcoxon matched-pairs test (*, *P* < 0.05; n.s., not significant).

The Δ*ytfE* single mutant strain had reduced fitness relative to the WT strain (Fig. 2); however, the single Δ*ytfE* mutant was not significantly more sensitive than the WT strain to the presence of acidified nitrite during growth in culture. Similarly, despite the lowered fitness of the Δ*hmp* Δ*ytfE* strain in spleens relative to the Δ*hmp* strain, the Δ*hmp* Δ*ytfE* strain was not significantly more sensitive than the Δ*hmp* mutant strain to the presence of acidified nitrite during growth in culture (Fig. 4A, compare +NO_2_ samples). The absence of *ytfE* also did not significantly alter the sensitivity of strains to the NO donor compound DETA-NONOate (diethylenetriamine NONOate) during growth in minimal medium (Fig. 4B). This is consistent with previous reports on other organisms (38, 39), perhaps due to the presence of other backup repair pathways that are active in the absence of Hmp or because YtfE plays a role in protection from other stress species.

We then compared *hmp* reporter expression levels in Δ*ytfE* and Δ*hmp* Δ*ytfE* strains to confirm that NO diffusion occurred across Δ*hmp* Δ*ytfE* microcolonies using plasmid-borne reporters. Mice were infected intravenously with Δ*ytfE* GFP^+^ *P_hmp_*::*mCherry* or Δ*hmp* Δ*ytfE* GFP^+^ *P_hmp_*::*mCherry* strains, and spleens were harvested at 3 days p.i. to visualize reporter expression by fluorescence microscopy. The Δ*ytfE* strain had peripheral *P_hmp_* reporter expression, as seen with the chromosomally integrated *hmp* reporter, indicating that NO diffusion across the microcolony is inhibited by peripheral cells in Δ*ytfE* microcolonies (Fig. 4C and D). Δ*hmp* Δ*ytfE* microcolonies showed no such preference for the periphery, indicating that NO diffused across these centers, as expected based on the loss of *hmp*.

### Rescue of *ytfE* restores microcolony size.

**FIG 5:**
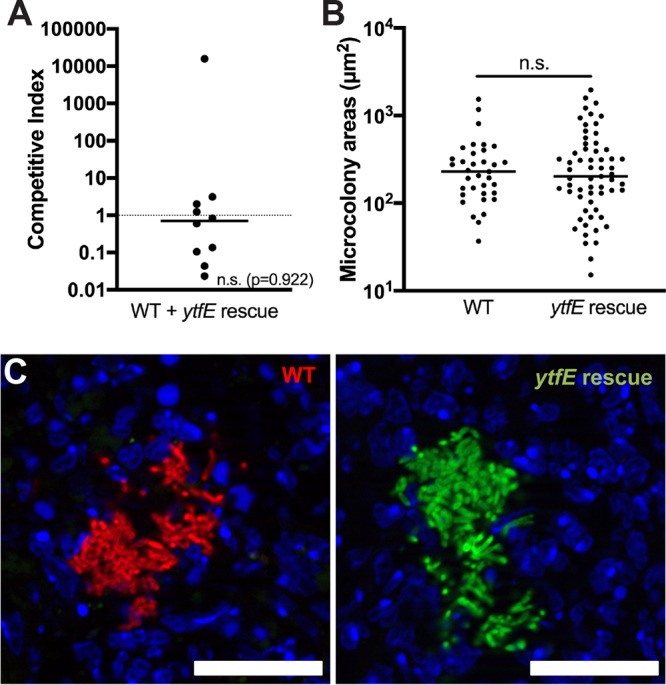
Rescue of *ytfE* restores fitness of the Δ*ytfE* strain. (A) C57BL/6 mice were inoculated i.v. with an equal mixture of *ytfE*-rescued GFP^+^ and WT mCherry^+^ strains, and spleens were harvested at day 3 p.i. The competitive index is the ratio of *ytfE* rescue/WT CFU in the spleen at day 3 divided by the ratio of *ytfE* rescue/WT CFU in the inoculum. Dots represent individual mice. The dotted line indicates equal fitness, with replicates completed on two separate days. Statistical analyses were performed by a Wilcoxon signed-rank test, compared to a value of 1 (n.s., not significant). (B) Quantification of WT and *ytfE* rescue microcolony areas (square micrometers) from coinfection (see Materials and Methods) in 10 mice. Dots represent individual microcolonies. (C) Representative images of WT mCherry^+^ and *ytfE*-rescued GFP^+^ microcolonies. Bars, 20 μm. Statistical analyses were performed by a Mann-Whitney test.

To show that the loss of *ytfE* was responsible for the decreased microcolony size, we rescued the Δ*ytfE* strain with a WT copy of *ytfE* and transformed the *ytfE*-rescued strain with the constitutive *gfp*-expressing plasmid. Mice were infected intravenously with WT mCherry^+^ and *ytfE*-rescued GFP^+^ strains, and spleens were harvested at day 3 p.i. to quantify CFU and visualize microcolony areas by fluorescence microscopy. The median competitive index for the *ytfE*-rescued strain was close to a value of 1, indicating that the fitness of this strain was roughly equivalent to that of the WT strain (Fig. 5A). The microcolony areas were quantified within all the spleens depicted in Fig. 5A, and the areas of *ytfE*-rescued microcolonies were very similar to those of WT microcolonies (Fig. 5B and C). These results indicate that the Δ*ytfE* strain was rescued by the WT copy of *ytfE*, which confirms that the reduced fitness of the Δ*ytfE* strain was specifically due to a loss of *ytfE*.

## DISCUSSION

The detoxification of NO and other reactive nitrogen species is critical for bacterial survival within host tissues (40–43). Bacterial proteins involved in NO detoxification, however, are not synthesized until NO accumulates and damages the Fe-S clusters of NsrR, resulting in inactivation of this repressor (15, 16). Many additional Fe-S cluster-containing proteins are simultaneously damaged, so bacteria need to either repair damaged proteins or synthesize replacement proteins while simultaneously replenishing proteins that detoxify NO and prevent further damage (17, 38). Although Fe-S cluster repair is likely required for bacterial survival, it has been unclear whether or not this plays an important role within host tissues. We have chosen to ask this question in a mouse model of Y. pseudotuberculosis infection, where it is known that bacteria respond to reactive nitrogen species (RNS) and that Fe concentrations are limiting (24, 25, 32). *ytfE* expression had been detected in *Yersinia* species replicating within host tissues; however, it was unclear if Fe-S cluster repair or assembly plays a significant role during infection (36, 37). Here we have shown that Fe-S cluster repair contributes to successful Y. pseudotuberculosis replication within the spleen.

We found that a place where Fe-S cluster repair in Y. pseudotuberculosis likely occurs is in the peripheral subpopulation of bacteria that responds to NO assault within microcolonies. This is consistent with a role for YtfE in supporting survival of the peripheral subpopulation. In the absence of *ytfE*, we would expect bacteria on the periphery to be exposed to stress associated with NO exposure, leading to sequential loss of the peripheral population and progressively smaller microcolonies. Consistent with this hypothesis, we observed that Δ*ytfE* microcolonies were smaller than those established by the WT strain. It is expected that the difference between WT and Δ*ytfE* microcolony areas will become progressively more pronounced as the infection proceeds, because Δ*ytfE* microcolonies should continuously lose their peripheral subpopulation. This prediction is based on our previous observation that NO-sensitive microcolonies are progressively reduced during the course of disease, with elimination of Δ*hmp* bacteria by NO being most pronounced at late time points postinoculation, concurrent with a time point at which the animals inoculated with the WT strain are moribund (32).

NO alone was not sufficient to limit the growth of Δ*ytfE* bacteria in bacteriological medium, consistent with previous studies that indicated that the uropathogenic E. coli (UPEC) Δ*ytfE* strain had reduced intracellular survival within host cells but was not sensitive to exogenous NO alone (39). Presumably, the Δ*ytfE* strain is sensitive to other antimicrobial compounds generated within host tissues, possibly by the generation of a variety of RNS as a consequence of NO reaction with reactive oxygen species (ROS) or other compounds. Upregulation of *ytfE* following hydrogen peroxide-mediated damage of Staphylococcus aureus indicates that YtfE may play a broad role in repair, instead of just a response to nitrogen stress (44, 45). Additional studies in E. coli also suggest that YtfE repairs Fe-S cluster proteins damaged by hydrogen peroxide (13, 46–49).

An additional regulatory signal for the *ytfE* gene is iron limitation, which depresses the synthesis of new Fe-S cluster proteins, thus requiring cellular YtfE function (14, 21, 50). The spleen is expected to be an iron-limiting environment requiring Fe-S cluster repair, but we found no evidence for NO-independent induction of the *ytfE* gene within the center of microcolonies, using our fluorescent promoter constructions. This contrasts with data arguing that Y. pseudotuberculosis expresses many Fe acquisition genes during growth in host tissues, consistent with Fe-limiting conditions within host tissues (25, 37). It is possible that the center of the microcolony represents a protected environment with low exposure to stresses such as reactive nitrogen and oxygen species as well as limited damage to Fe-S centers. Additionally, *ytfE* expression may be induced at very low NO concentrations, as seen in Salmonella enterica serovar Typhimurium (38), but may require severe iron limitation for expression in the absence of NO.

The loss of YtfE function also has the potential to alter metabolite levels due to disruption of protein functions that are Fe-S center related. In the presence of RNS, a Δ*ytfE* strain may have reduced aconitase and fumarase activities due to the role of YtfE in Fe-S cluster repair specifically for these proteins (47). The Fe-S cluster of the NsrR repressor is also repaired by YtfE, which could lead to prolonged expression of the NsrR regulon in the presence of NO (13). Our results indicate that expression of the NsrR regulon is similar in the presence and absence of *ytfE*, although it remains possible that prolonged expression of the NsrR regulon could be detected at later time points postinoculation. Future work will investigate these issues and determine the interplay between iron regulation, NO-induced damage, and repair of critical Fe-S centers.

## MATERIALS AND METHODS

### Bacterial strains and growth conditions.

The WT Y. pseudotuberculosis strain YPIII was used throughout. For all mouse infection experiments, bacteria were grown overnight to postexponential phase in 2× YT broth (LB with 2× yeast extract and tryptone) at 26°C with rotation. Exponential-phase cultures were subcultured 1:100 from cultures grown overnight and grown at 26°C with rotation for an additional 2 h. Sodium nitrite (2.5 mM) was added to LB at pH 5.5 to induce the nitrogen stress response. The NO donor compound DETA-NONOate (2.5 mM) (Cayman Chemicals) was added to M9 minimal medium to test NO sensitivity.

### Generation of *ytfE* mutant strains.

The *hmp* and *nsrR* deletion strains were previously described (32). Deletion-derivative strains were generated for *ytfE* by amplifying the start codon plus 3 downstream codons and the 3′-terminal 3 codons plus the stop codon and fusing these fragments to generate a start plus 6-amino-acid plus stop construct. Deletion constructs were amplified with 500-bp flanking sequences on each side, cloned into the suicide vector pSR47S, and transformed into Y. pseudotuberculosis. Sucrose selection was used to select for bacteria that had incorporated the desired mutation after a second cycle of recombination (31). PCR, sequencing, and qRT-PCR were used to confirm deletion strains.

### Integrated *ytfE* reporter construction (*ytfE^+^* rescue strain).

The *ytfE*::*gfp* reporter was generated by cloning *gfp* immediately downstream of the *ytfE* gene (between the *ytfE* stop codon and terminator sequence) by overlap extension PCR. The *ytfE* start codon was amplified with 500 bp of upstream flanking sequence, while the stop codon of *gfp* was amplified with 500 bp of downstream flanking sequence. This fragment was cloned into the suicide vector pSR47S and transferred by conjugation into WT Y. pseudotuberculosis *hmp*::*mCherry* (chromosomally integrated), selecting for kanamycin resistance. For the *ytfE^+^* rescue strain, a WT *ytfE* gene product, including 500 bp upstream and downstream of *ytfE*, was amplified from genomic DNA and cloned into pSR47S. This vector was transferred by conjugation into Δ*ytfE* Y. pseudotuberculosis, selecting for kanamycin resistance. A second round of recombination was selected on sucrose-containing medium to isolate strains that had recombined each *ytfE* construct. PCR and sequencing were used to confirm the integration of *gfp* or rescue of the *ytfE* deletion.

### Generation of plasmid-based reporter strains.

Several of the Y. pseudotuberculosis reporter strains in this study have been previously described: WT GFP^+^, WT mCherry^+^ (*yopE*::*mCherry*), WT *hmp*::*mCherry*, WT GFP^+^ *hmp*::*mCherry*, and Δ*hmp* GFP^+^ (32). For this study, GFP^+^ strains were constructed by transforming deletion strains with the constitutive GFP plasmid, which expresses GFP from an unrepressed *P_tet_* promoter of pACYC184. *P_hmp_*::*mCherry* was also transformed into GFP^+^ strains. The *P_hmp_*::*mCherry* transcriptional fusion was previously constructed by fusing the *hmp* promoter to *mCherry* using overlap extension PCR and cloned into the pMMB67EH plasmid (32).

### Murine model of systemic infection.

Six- to eight-week-old female C57BL/6 mice were obtained from Jackson Laboratories (Bar Harbor, ME). All animal studies were approved by the Institutional Animal Care and Use Committee of Tufts University. Mice were injected intravenously with 10^3^ bacteria for all experiments. For coinfection experiments, mice were inoculated with 5 × 10^2^ CFU of each strain, for a total of 10^3^ CFU. At the indicated time points postinoculation (p.i.) (3 days), spleens were removed and processed.

### qRT-PCR to detect bacterial transcripts in broth-grown cultures.

Bacterial cells were grown in broth to an *A*_600_ of 0.3 under appropriate stress conditions, pelleted, and resuspended in RLT buffer (Qiagen) plus β-mercaptoethanol, and RNA was isolated using the RNeasy kit (Qiagen) according to the manufacturer’s protocol. DNA contamination was eliminated using the DNA-free kit (Ambion) according to the manufacturer’s protocol. RNA was reverse transcribed using Moloney murine leukemia virus (M-MLV) reverse transcriptase (Invitrogen), in the presence of the RNase inhibitor RNaseOut (Invitrogen), according to the manufacturer’s protocol. Approximately 30 ng cDNA was used as a template in reactions with 0.5 μM forward and reverse primers and SYBR green (Applied Biosystems) according to the manufacturer’s protocol. Control samples that lacked M-MLV were prepared, to confirm that DNA was eliminated from samples and was not amplified by qRT-PCR. Reactions were carried out using the StepOnePlus real-time PCR system, and relative comparisons were obtained using the ΔΔ*C_T_* or $2^{-\Delta C_{T}}$ method (Applied Biosystems).

### qRT-PCR to detect bacterial transcripts from mouse tissues.

Mice were inoculated intravenously with the WT strain, and at day 3 p.i., spleens were harvested and immediately submerged in RNAlater solution (Qiagen). Tissue was homogenized in RLT buffer plus β-mercaptoethanol, and RNA was isolated using the RNeasy kit (Qiagen) according to the manufacturer’s protocol. Bacterial RNA was enriched following depletion of host mRNA and rRNA from total RNA samples, using the MICROBEnrich kit (Ambion) according to the manufacturer’s protocol. DNA digestion, reverse transcription, and qRT-PCR were performed as described above.

### Fluorescence microscopy.

C57BL/6 mice were inoculated intravenously with the Y. pseudotuberculosis WT strain, and at day 3 p.i., spleens were harvested and immediately fixed in 4% paraformaldehyde in phosphate-buffered saline (PBS) for 3 h. Tissues were frozen-embedded in Sub Xero freezing medium (Mercedes Medical) and cut by a cryostat microtome into 10-μm sections. To visualize reporters, sections were thawed in PBS, stained with Hoechst stain at a 1:10,000 dilution, and washed in PBS, and coverslips were mounted using ProLong gold (Life Technologies). Tissue was imaged with a 20× or 63× objective, using a Zeiss Axio Observer.Z1 fluorescence microscope (Zeiss) with a Colibri.2 LED light source, Apotome.2 (Zeiss) for optical sectioning, and an Orca-R^2^ digital charge-coupled-device (CCD) camera (Hamamatsu).

### Image analysis.

Volocity image analysis software was used to quantify microcolony areas. ImageJ was used to quantify the signal intensity of each channel at the centroid and periphery of each microcolony, to generate relative signal intensities of fluorescent reporters. Thresholding was used to define the area of each microcolony, the centroid was calculated, and 0.01 pixel^2^ squares were selected to calculate values at the centroid. Peripheral measurements depict bacteria in contact with host cells.
